# Care gaps among people presenting to the hospital following self-harm: observational study of three emergency departments in England

**DOI:** 10.1136/bmjopen-2024-085672

**Published:** 2024-10-22

**Authors:** Sarah Steeg, Harriet Bickley, Caroline Clements, Leah M Quinlivan, Steven Barlow, Elizabeth Monaghan, Fiona Naylor, Jonathan Smith, Faraz Mughal, Catherine Robinson, Shamini Gnani, Navneet Kapur

**Affiliations:** 1 NIHR School for Primary Care Research, University of Manchester, Manchester, UK; 2 Manchester Academic Health Science Centre, University of Manchester, Manchester, UK; 3 Centre for Mental Health and Safety, Division of Psychology and Mental Health, School of Health Sciences, University of Manchester, Manchester, UK; 4 NIHR Greater Manchester Patient Safety Research Collaboration, School of Health Sciences, University of Manchester, Manchester, UK; 5 School of Medicine, Keele University, Keele, UK; 6 Social Care and Society, School of Health Sciences, University of Manchester, Manchester, UK; 7 Department of Primary Care and Public Health, Imperial College London, London, UK; 8 Mersey Care NHS Foundation Trust, Liverpool, UK

**Keywords:** MENTAL HEALTH, Patients, Suicide & self-harm, PUBLIC HEALTH, Psychosocial Intervention, Emergency Departments

## Abstract

**Abstract:**

**Objectives:**

This study aims to examine the proportions of patients referred to mental health, social and voluntary, community and social enterprise (VCSE) services and general practice and to assess care gaps among people presenting to the hospital following self-harm.

**Design:**

Population-based observational study. Data were extracted from hospital records.

**Setting:**

Three emergency departments (EDs) in Manchester, UK.

**Participants:**

26 090 patients aged 15+ years who presented to participating EDs following self-harm and who received a psychosocial assessment by a mental health specialist.

**Primary and secondary outcome measures:**

Primary outcome measures are as follows: care gaps, estimated from the proportion of patients with evidence of social and mental health needs with no new or active referral to mental health, social and VCSE services. Secondary outcome measures are as follows: proportions of referrals by groups of patients, estimated mental health and social needs of patients. Indicators of mental health and social need were developed with academic clinicians (psychiatrist, general practitioner and social worker) and expert lived experience contributors.

**Results:**

96.2% (25 893/26 909) of individuals were estimated as having mental health needs. Among this group, 29.9% (6503/21 719) had no new or active referral to mental health services (indicating a care gap). Mental healthcare gaps were greater in men and those who were aged under 35 years, from a black, South Asian or Chinese ethnic group, living in the most deprived areas and had no mental health diagnosis, or alcohol, substance misuse, anxiety or trauma-related disorder. 52.8% (14 219/26 909) had social needs, with care gaps greater for men, individuals aged 45–64 and those who were unemployed or had a diagnosed mental disorder.

**Conclusions:**

Care gaps were higher among hospital-presenting groups known to have increased risks of suicide: men, those in middle age, unemployed individuals and those misusing substances. Improved access to mental health, social and VCSE services and general practice care is vital to reduce inequities in access to self-harm aftercare.

**STRENGTHS AND LIMITATIONS OF THIS STUDY**

- The use of a self-harm cohort study allowed a detailed assessment of patients’ needs and referrals to mental healthcare, social and voluntary, community and social enterprise services and general practitioners.
- Measures of mental health and social needs were codeveloped with lived experience contributors, researchers and clinicians.
- Our study could not include people not receiving a psychosocial assessment by a mental health specialist because information relating to mental health and social needs was not available in this group.
- The use of validated measures would have provided more accurate and nuanced estimates of mental health and social needs; for example, we were unable to estimate severity of needs or discern the level of impairment to daily activities.

## Introduction

 People who present to the hospital following self-harm are a priority group for suicide prevention due to their increased risk of suicide.^1^ Self-harm includes intentional self-poisoning or self-injury and can involve varying degrees of suicidal intent.^2^ Appropriate aftercare for people who present to the hospital following self-harm is central to suicide prevention. However, few studies have examined care gaps in this population. While studies to date have examined clinical management of self-harm in different groups, none have specifically linked referral rates to levels of need—thus enabling estimation of care gaps. The roles of social and voluntary, community and social enterprise (VCSE) services and general practitioner (GP) care are also under-researched.

There is widespread recognition that care for people who have self-harmed should be multiagency and interdisciplinary; many people who have harmed themselves face social and economic adversities that exacerbate mental health problems.^3 4^ Guidance from the National Institute for Health and Care Excellence on the management and prevention of self-harm, therefore, recommends joint approaches between social care agencies, healthcare professionals and VCSE services.^2^ In addition, the latest suicide prevention strategy for England, launched in 2023, highlights the pivotal role of VCSE services in suicide prevention, calling for strong collaboration with health and local government services.^1^

Much of the research into self-harm aftercare to date has focused on psychosocial assessments and psychological therapies.^5^,^9^ Little attention has been given to the role of social services, VCSE organisations and primary care. For example, there has been very little research into social work-based or integrated interventions for preventing suicide^10 11^ or the role of voluntary-sector-led support.^12^ Similarly, while general GPs have a pivotal role in reviewing patients’ needs and linking with VCSE organisations following self-harm,^13^ most studies of clinical management have not considered referrals to GPs.

It is vital to recognise people’s wider psychosocial needs when considering care gaps in populations of people experiencing poor mental health.^14^ While care gaps have been examined in general population samples and among people with specific mental disorders,^15^,^18^ there has been no assessment of care gaps for those seeking help after self-harm. The terms ‘healthcare needs analysis’, ‘treatment gaps’ and ‘care gaps’ all focus on incidence/prevalence rates of disease, provision of appropriate care and differences between groups. In the present study, we use the term ‘care gaps’; this concept has been recommended as more appropriate for mental health as it takes into account non-clinical interventions and psychosocial needs.^14^ Without a comprehensive analysis of needs, the potential effectiveness of psychological treatments for self-harm may be compromised. For example, evidence for the effectiveness of psychological interventions for self-harm is relatively weak despite a large body of research spanning decades.^19^

Routine sources of health and social data are valuable in examining care gaps.^14 20^ Most national register studies used to examine suicidal behaviour do not contain key information such as specific life events preceding a self-harm episode.^21^ However, dedicated, health condition-specific cohort studies contain more relevant information than national, service-wide health data. Using data from the Manchester Self-Harm Project, we examined the likelihood of referrals to mental health and social care services and to VCSE organisations for people attending hospital following self-harm, and their mental health and social needs.

Our specific research objectives were as follows:

1. To describe proportions of mental health, social and VCSE services and GP referrals among a cohort of people presenting to the hospital following self-harm.
2. To compare frequencies and probabilities of referrals between groups of patients, including age, gender, employment status, existing mental health diagnosis, ethnic and area-level deprivation groups.
3. To estimate mental health and social needs among groups of patients including age, gender, employment status, existing mental health diagnosis, ethnic and area-level deprivation groups.
4. To describe proportions referred to mental health, social and VCSE services and GP by prevalence of social and mental health need, thus estimating care gaps (primary outcome measure).

## Methods

### Study design and data sources

Data from the Manchester Self-Harm Project, a prospective cohort study of people presenting to emergency departments (EDs) in Manchester, UK, were used in this study. The Manchester Self-Harm Project includes approximately 65 000 episodes of self-harm by around 37 000 people presenting to three EDs between 1997 and 2017. The study includes episodes of intentional self-poisoning or self-injury, regardless of motivation. A range of demographic, clinical and area-based data were collected from ED and mental health service records, following each presentation involving self-harm. Research administrators used validated search terms to identify presentations potentially involving self-harm. Where self-harm was confirmed, data were extracted using a two-stage process. First, basic clinical and demographic data (including reason for attendance, method of self-harm, age, gender and ethnic group) were extracted from ED records for all episodes. Second, further information was extracted from psychosocial assessments for episodes that were assessed by a mental health specialist. In this stage, researchers coded the information in the written records of the assessments using a standard proforma and following a protocol. If uncertainty arose during coding, the research team discussed the anonymised case to reach a consensus. Accuracy and inter-rater reliability were assessed using a period of training for all researchers, including coding a random selection of assessments independently and then comparing codes within the research team. This helped to identify areas of inconsistency and inaccuracy in applying coding rules. Validation exercises of the proformas against clinical records have shown high levels of agreement (κ ⩾ 0.8 for individual variables).^6^ Variables added during this stage included time of self-harm, suicidal intent (yes/no), suicide note, evidence of preplanning, concealment of self-harm, history of drug or alcohol misuse, psychiatric diagnosis, history of self-harm, current and previous mental health service involvement, current symptoms of depression, factors identified by the patient as precipitating the self-harm (eg, problems with relationships, family, housing, work, school, money, mental health, physical health, abuse, legal issues, being a victim of crime, drug or alcohol misuse and miscarriage) and clinical management (eg, referral, admission and discharge).

We analysed individuals rather than episodes due to many of the exposure characteristics (eg, gender, age, ethnic group and mental health diagnosis) being measured at an individual level. In addition, mental healthcare gaps are typically measured at the individual level^14^; including multiple episodes by the same individual would likely lead to an inaccurate estimation of care gaps. Where there were multiple episodes by the same individual, the individual’s first assessed episode during the study period was included.

The study protocol was preregistered (https://osf.io/zq5et). Following preliminary data analysis, it was apparent that the data relating to physical health problems was only available for people who had reported physical health as a direct precipitant to the self-harm. This was likely to be an underestimate of the prevalence of physical health problems in the cohort. Therefore, our study deviated from the planned protocol by focusing on mental health and social needs. This study followed the Strengthening the Reporting of Observational Studies in Epidemiology guidelines for reporting observational cohort studies.^22^

#### Clinical management (secondary outcome measures)

**Figure 1:**
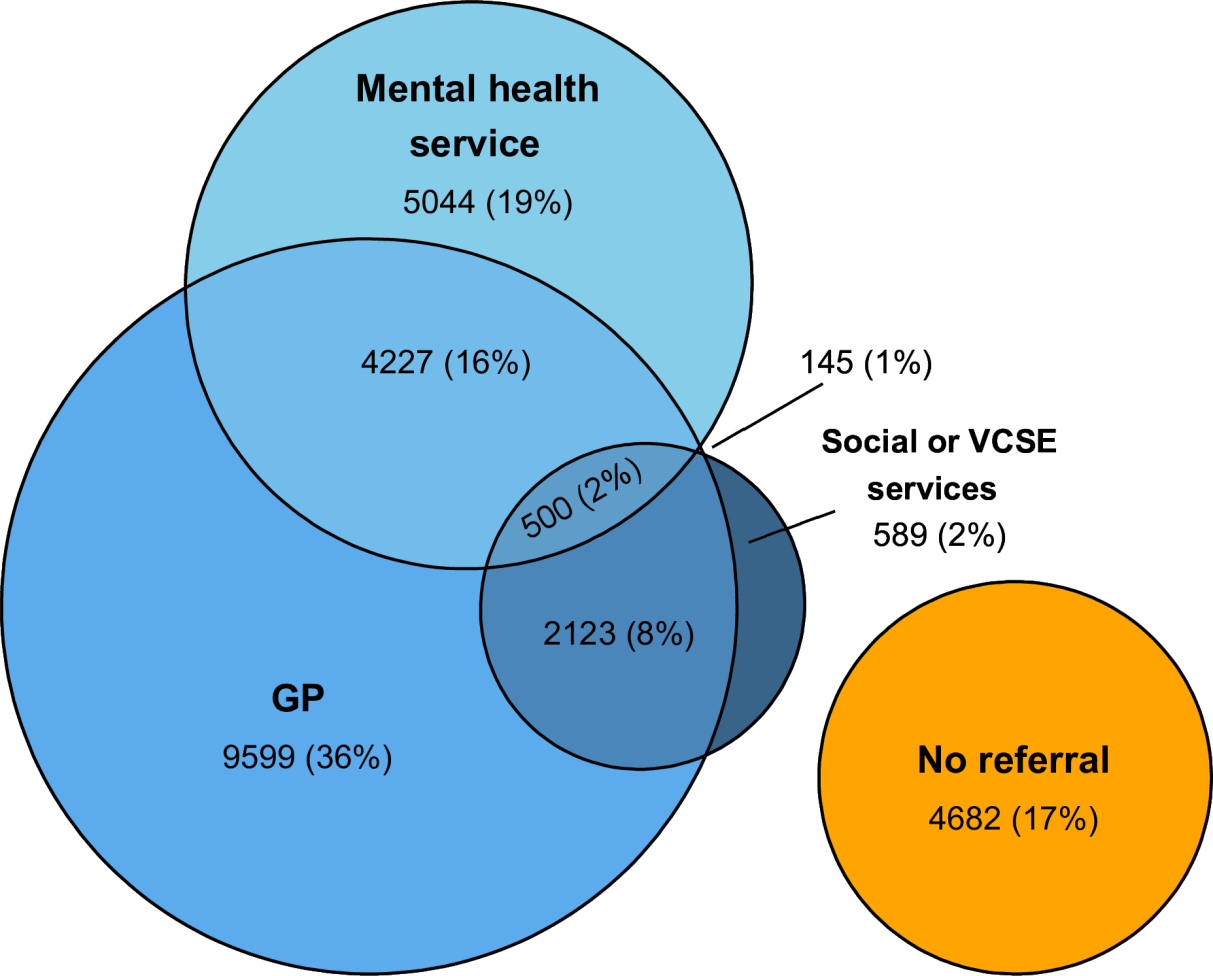
Venn diagram showing percentages of patients referred to their GP, to mental health services and to social or VCSE services following hospital presentation for self-harm. GP, general practitioner; VCSE, voluntary, community and social enterprise.

We examined the following categories of clinical management: referral to mental health services (including referral to outpatient mental health follow-up, crisis or urgent care services, community mental health services and drug and alcohol services), referral to social services, referral to VCSE services and referral to general practice (including recommendations for the GP to refer for primary mental healthcare). We only included formal referrals and did not include instances where the patient was advised to self-refer. Individuals could be referred to more than one service for the same episode of self-harm (figure 1).

#### Care gaps (primary outcome measures)

Referral to mental health, social and VCSE services and GP following self-harm and characteristics pertaining to patients’ mental health and social needs were assessed using information recorded in hospital notes and specialist mental health assessments (table 1). Data from psychosocial assessments were used to make inferences about mental health and social needs. The indicators were devised in the context of a clinical population of people who had presented to ED with self-harm. For example, if a life event such as a financial problem was mentioned in the psychosocial assessment as a contributing factor to the self-harm, this was interpreted as a substantial social problem. Given the absence of validated measures of mental health and social needs in this population, indicators of mental health and social services/VCSE sector needs were codeveloped with researchers, clinicians (an academic clinical psychiatrist, an academic GP and an academic social worker) and an expert lived experience panel comprising four people with personal experience of attending ED for self-harm as a patient or carer. The codevelopment process involved an initial meeting to discuss the factors available in the study that may indicate mental health or social needs, followed by an exercise where each expert was asked to specify which factors should be included as indicating mental health needs and which may indicate social needs. There was broad agreement between the experts. In instances where consensus was not reached in the initial selection of factors, the lead author facilitated further discussion. Two measures were derived:

1. Evidence of mental healthcare needs, derived from the presence of any of the following: any mental health diagnosis, current drug or alcohol misuse, self-harm that was reported as directly in response to mental symptoms or a mental disorder, the presence of a suicide note, patient reporting that they wanted to die at the time of the self-harm and symptoms of depression (table 1).
2. Evidence of significant social problems, derived from homelessness or hostel dwelling, self-harm in response to problems with housing, money, work or study, or in response to legal problems or physical, sexual or emotional abuse (table 1).

**Table 1:** Variables used to derive measures of mental health and social needs

Patient characteristics	Mental healthcare needs	Significant social needs
Homeless or living in hostel accommodation		**✓**
Currently misusing alcohol	**✓**	
Currently misusing drugs	**✓**	
Has a mental health diagnosis	**✓**	
Precipitants of self-harm or cause(s) of current distress		
Housing problem		**✓**
Employment or study problems		**✓**
Legal problem, for example, criminal charges		**✓**
Victim of crime		**✓**
Financial problems		**✓**
Direct response to mental symptoms	**✓**	
Other mental health problems	**✓**	
Abuse (physical, mental, sexual)	**✓**	**✓**
Alcohol abuse	**✓**	
Substance abuse	**✓**	
Circumstances of the self-harm		
Suicide note	**✓**	
Intention to die during attempt	**✓**	
Symptoms of depression		
Suicidal thoughts	**✓**	
Suicidal plans	**✓**	
Hallucinations/delusions	**✓**	
Looks depressed	**✓**	
Feels depressed	**✓**	
Sleep disturbance	**✓**	
Appetite disturbance	**✓**	
Feels hopeless	**✓**	
Low energy	**✓**	
Evidence of hostility	**✓**	
Any mental health diagnosis	**✓**	

Individuals were defined as having mental healthcare needs met if they were currently receiving mental healthcare or were referred to mental health services following their hospital presentation for self-harm. Significant social needs were defined as being met if the individual was referred to social services or VCSE services. The measures of clinical management (new and existing referrals) and the codeveloped measures of mental health/social needs were used to estimate care gaps, which were defined as ‘the percentage of individuals who require care but do not receive treatment’ as described by Kohn *et al*,^23^ with the term ‘treatment’ encompassing existing care and new referrals to care made following the hospital presentation.

#### Study covariates

In addition to overall estimates, we examined estimates stratified by gender and age groups, presence of existing mental health diagnosis, ethnic groups and area-level deprivation quintile. The specific age groupings were determined based on the size of the outcome groups. Likewise, mental health diagnosis groupings were collapsed to enable analysis when there were too few patients in a single diagnostic category. Ethnic group categories were based on the Office for National Statistics 2011 census broad groupings. In subgroup analyses where numbers were too low to report findings (<10), we suppressed cell counts and estimates for the specific ethnic group. This enabled us to retain broad groupings rather than collapsing ethnic minority groups into a single category. Mental health diagnosis categories used were mood disorders (including depression and bipolar disorder), anxiety and trauma-related disorders (including anxiety and post-traumatic stress disorder), psychotic disorders (including schizophrenia), eating disorders, personality disorders, alcohol dependence, substance abuse, multisubstance abuse and learning difficulties or autism). We also included separate groups for alcohol misuse and substance misuse. Diagnoses were based on International Classification of Diseases 10th Revision (ICD-10) codes.

### Missing data

Factors used to estimate mental health and social needs (including demographic characteristics, precipitants to and circumstances of the self-harm, symptoms of depression) and categories of clinical management were coded as absent if there was no record of them in the psychosocial assessment. Missing data on age, sex and ethnic group were imputed using data from any additional episodes from the same individual recorded in the Manchester Self-Harm Project dataset. Data on exposure variables were missing for between 0% and 6% of individuals. No individuals had missing data for age, three individuals were excluded due to missing data on gender and missing data for other variables were excluded pairwise to maximise the cohort size: 565 (2.1%) had missing ethnic group data, 1499 (5.6%) had missing employment status data and 1171 (4.4%) had missing area-level deprivation data. There were no substantial differences in outcome measures between patients with and without missing exposure data (online supplemental table S1).

### Study sample

Our primary study sample for objectives 1–3 was 26 909 individuals: all patients aged 15 years or over presenting between 1997 and 2017, with data available on gender (n=3 were missing) and who received a psychosocial assessment (n=12 174 received no assessment). Our primary study cohorts for objective 4 were patients assessed by the research team as having significant mental health (N=25 893) or social (N=14 219) needs. In adjusted analyses, we restricted these cohorts to individuals with data available for confounding variables (N=21 719 and 11 892, respectively).

### Statistical analysis

Frequencies of health and social care referrals were estimated as a proportion of the broader study sample. Proportions and their 95% CIs are presented. Log binomial regression models were used to estimate probability (risk) ratios of referrals to mental health and social care services among gender and age groups, presence of existing mental health diagnoses, ethnic groups and area-level deprivation quintiles. Risk ratios with CIs above 1.0 indicated an exposure was associated with an increased probability of referral in that group compared with the reference group. The following reference groups were used in the regression models: women, aged 65+, white ethnic group, in work or study, the least deprived Index of Multiple Deprivation (IMD) quintile and the group with no psychiatric diagnosis. Unadjusted and adjusted risk ratios (aRRs) were estimated, with models adjusted for factors known to be associated with referral likelihood: year of presentation, hour of presentation, hospital attended, role of assessor (doctor or nurse) and method of self-harm.

### Patient and public involvement

An expert lived experience panel, comprising four individuals with personal experience of attending an ED for self-harm, was involved in designing the study, developing measures of mental health and social needs (see ‘Assessing clinical management and mental health and social needs of patients’) and in interpreting the findings of the study.

## Results

### Characteristics of the cohort

26 909 individuals presented with self-harm between 1997 and 2017 and received a psychosocial assessment. Three individuals were excluded due to missing data on gender. There were no individuals with missing data for age. Proportions of missing data for other exposure variables were between 2% and 6% (online supplemental table S1). 55.8% (15019/26909) of the cohort was female, 32.7% (8805) were aged under 25 years and 1.6% (419) were aged 65 years or over. 88.9% (23421) of the cohort were from a white ethnic group, 4.5% (1193) were from an Indian/Pakistani/Bangladeshi background, 2.6% (695) were from a black African/Caribbean ethnic group, 617 (1.6%) were from a mixed ethnic group, 0.3% (116) were Chinese and 1.4% (564) were from another ethnic group. The most deprived quintile (n=5408) within the cohort lived in areas with a mean rank of 421 (out of 32 482 lower super output areas), while the least deprived quintile (n=4959) had a mean rank of 19 613/32 482. Therefore, the least deprived quintile within this cohort was broadly within the most deprived 60% of areas nationally.

52.5% (14163) of the cohort had a mental health diagnosis recorded; 16.5% (4445) mood disorder, 10.1% (2706) alcohol use disorder (defined as daily alcohol use of 7 units or more), 4.9% (1305) had alcohol dependence, 5.3% (1416) had anxiety or trauma-related disorder, 4.6% (1225) were misusing substances or had a substance use disorder (an additional 3.3%, 888, had multisubstance misuse disorder), 4.2% (1133) were diagnosed with a personality disorder, 2.3% (613) had a psychotic disorder and 0.7% (191) had an eating disorder. In addition, 0.9% (241) had learning difficulties or autism.

### Clinical management

Overall, 36.9% (9916) of patients in the cohort were referred to mental health services: 13.2% (3542) to outpatient mental health services (table 2), 9.8% (2623) crisis or urgent care, 4.0% (1072) to alcohol and drug services and 3.5% (948) to community mental health services (online supplemental table S2). 1.5% (393) were referred to social services and 11.3% (3047) were referred to VCSE services (table 2). Referral to more than one service was common (figure 1). Groups more likely to be referred to mental health services included men, older age groups, those who were unemployed, registered sick or retired and those with a mental health diagnosis (table 2). The youngest (15–19 years) and oldest (65+ years) age groups were most likely to be referred to social services, as were people living in more deprived areas. Younger age groups and those with a diagnosis of anxiety and trauma-related disorders were most likely to be referred to VCSE services (table 2). Overall, 61.1% (16 449) were referred to their GP. For one-fifth of individuals (19.9%, 5357), a GP referral was only a new or current referral in place. This proportion was higher for younger people (ages 15–19, 25.1%, 95% CI 23.8% to 26.5%), black (25.8%, 95% CI 22.6% to 29.1%) and South Asian (27.2%, 95% CI 24.7% to 30.0%) people and those with no mental health diagnosis (26.2%, 95% CI 25.4% to 27.0%).

**Table 2:** Proportions of patients referred to mental health, social and VCSE services and their GP (objectives 1 and 2)*

	%, 95% CI (n) referred to mental health services	%, 95% CI (n) referred to social services	%, 95% CI (n) referred to VCSE services	%, 95% CI (n) referred to GP	%, 95% CI (n) referred to GP with no other new referral or current mental healthcare
Total (26 909)	36.9, 36.3 to 37.4 (9916)	1.5, 1.3 to 1.6 (393)	11.3, 11.0 to 11.7 (3047)	61.1, 60.5 to 61.7 (16449)	19.9, 19.4 to 20.4 (5357)
Women (15 019)	35.5, 34.7 to 36.3 (5331)	1.7, 1.5 to 1.9 (257)	11.8, 11.3 to 12.3 (1771)	63.4, 62.6 to 64.2 (9521)	19.6, 18.9 to 20.2 (2936)
Men (11 890)	38.6, 37.7 to 39.4 (4585)	1.1, 1.0 to 1.4 (136)	10.7, 10.2 to 11.3 (1276)	58.3, 57.4 to 59.2 (6928)	20.4, 19.6 to 21.1 (2421)
Age group					
15–19 (3931)	30.9, 29.4 to 32.3 (1213)	2.1, 1.7 to 2.6 (82)	16.5, 15.4 to 17.7 (648)	62.9, 61.4 to 64.4 (2473)	25.1, 23.8 to 26.5 (986)
20–24 (4874)	33.4, 32.1 to 34.7 (1626)	1.2, 0.9 to 1.5 (57)	13.8, 12.9 to 14.8 (673)	61.3 59.9 to 62.6 (2987)	21.2, 20.1 to 22.4 (1035)
25–34 (6982)	38.1, 37.0 to 39.2 (2660)	1.4, 1.1 to 1.7 (95)	10.5, 9.8 to 11.3 (734)	60.5, 59.3 to 61.6 (4223)	19.5, 18.5 to 20.4 (1358)
35–44 (5749)	38.2, 37.0 to 39.5 (2196)	1.3, 1.1 to 1.7 (77)	9.7, 9.0 to 10.5 (560)	63.2, 61.9 to 64.4 (3633)	19.4, 18.4 to 20.5 (1117)
45–64 (4954)	39.8, 38.4 to 41.1) (1969)	1.4, 1.1 to 1.8 (69)	8.2, 7.4 to 9.0 (404)	59.8, 58.5 to 61.2 (2964)	16.4, 15.3 to 17.4 (810)
65+ (419)	60.1, 55.4 to 64.7 (252)	3.1, 1.8 to 5.3 (13)	6.7, 4.7 to 9.5 (28)	40.3, 35.7 to 45.1 (169)	12.2, 9.4 to 15.7 (51)
Ethnic group (26344)†					
White (23 421)	36.9, 36.3 to 37.5 (8648)	1.4, 1.3 to 1.6 (338)	11.3, 10.9 to 11.8 (2655)	61.6, 61.0 to 62.2 (14434)	19.6, 19.1 to 20.1 (4582)
Black (695)	39.1, 35.6 to 42.8 (272)	1.7, 1.0 to 3.0 (12)	13.4, 11.0 to 16.1 (93)	60.6, 56.9 to 64.1 (421)	25.8, 22.6 to 29.1 (179)
Indian/Pakistani/Bangladeshi (1193)	34.0, 31.4 to 36.8 (406)	1.6, 1.0 to 2.5 (19)	10.0, 8.4 to 11.8 (119)	62.4, 59.6 to 65.1 (744)	27.2, 24.7 to 30.0 (324)
Mixed race (521)	41.1, 36.9 to 45.4 (214)	2.1, 1.2 to 3.8 (11)	9.8, 7.5 to 12.7 (51)	49.7, 45.4 to 54.0 (259)	14.6, 11.8 to 17.9 (76)
Chinese (73)	26.0, 17.3 to 37.2 (19)	−	−	50.7, 39.4 to 61.9 (37)	19.2, 11.7 to 29.8 (14)
Other (441)	36.1, 31.7 to 40.6 (159)	−	−	53.7, 49.1 to 58.3 (237)	19.3, 15.9 to 23.2 (85)
Employment status‡ (25 410)					
In work or study (9616)	31.3, 30.4 to 32.2 (3009)	0.8, 0.6 to 1.0 (76)	11.4, 10.8 to 12.1 (1097)	64.5, 63.7 to 65.6 (6221)	25.6, 24.8 to 26.5 (2464)
Unemployed (11 585)	39.9, 39.0 to 40.8 (4623)	1.7, 1.5 to 1.9 (195)	11.0, 10.4 to 11.6 (1272)	57.1, 56.2 to 58.0 (6614)	16.3, 15.7 to 17.0 (1892)
Registered sick (2504)	40.7, 38.8 to 42.6 (1019)	2.6, 2.0 to 3.3 (64)	15.2, 13.8 to 16.6 (380)	75.0, 73.3 to 76.7 (1879)	16.0, 14.6 to 17.5 (401)
Retired (613)	53.8, 49.9 to 57.7 (330)	2.6, 1.6 to 4.2 (16)	6.4, 4.7 to 8.6 (39)	49.3, 45.3 to 53.2 (302)	15.8, 13.1 to 18.9 (97)
Looking after the home or family/other (1092)	31.8, 29.1 to 34.6 (347)	2.2, 1.5 to 3.3 (24)	13.1, 11.2 to 15.2 (143)	73.8, 71.1 to 76.3 (806)	26.8, 24.3 to 29.5 (293)
Area-level deprivation (IMD) quintile (25 738)§¶					
1 (least deprived) (5065)	35.0, 33.7 to 36.3 (1773)	1.0, 0.8 to 1.3 (52)	9.4, 8.6 to 10.2 (474)	59.6, 58.8 to 61.0 (3020)	19.0, 18.9 to 20.1 (963)
2 (5178)	38.8, 37.5 to 40.2 (2010)	1.2, 0.9 to 1.5 (62)	11.6, 10.8 to 12.5 (602)	61.1, 59.7 to 62.4 (3163)	19.2, 18.2 to 20.3 (996)
3 (5151)	38.2, 36.9 to 39.5 (1968)	1.8, 1.5 to 2.2 (93)	11.0, 10.2 to 11.9 (568)	61.8, 60.4 to 63.1 (3181)	19.6, 18.6 to 20.7 (1011)
4 (5034)	39.0, 37.7 to 40.4 (1965)	1.5, 1.2 to 1.8 (74)	10.9, 10.1 to 11.8 (549)	61.4, 60.0 to 62.7 (3089)	19.8, 18.7 to 20.9 (996)
5 (most deprived) (5310)	34.2, 32.9 to 35.5 (1815)	1.6, 1.3 to 1.9 (83)	13.1, 12.2 to 14.1 (697)	63.8, 62.5 to 65.1 (3390)	22.0, 20.9 to 23.2 (1170)
Primary psychiatric diagnosis (26 909)					
None recorded (12 746)	29.8, 29.0 to 30.6 (3799)	1.5, 1.3 to 1.7 (187)	12.8, 12.3 to 13.4 (1636)	63.7, 62.9 to 64.6 (8122)	26.2, 25.4 to 27.0 (3339)
Mood disorder (4445)	49.2, 47.8 to 50.7 (2188)	1.5, 1.1 to 1.9 (65)	10.6, 9.8 to 11.6 (473)	59.1, 57.7 to 60.6 (2628)	10.2, 9.3 to 11.1 (453)
Psychotic disorder (613)	68.0, 64.2 to 71.6 (417)	-	6.7, 5.0 to 9.0 (41)	33.0, 29.3 to 36.8 (202)	-
Anxiety or trauma-related disorder (1416)	32.6, 30.2 to 35.1 (462)	1.6, 1.0 to 2.3 (22)	18.4, 16.5 to 20.5 (261)	74.4, 72.0 to 76.6 (1053)	23.7, 21.5 to 25.9 (335)
Eating disorder (191)	38.7, 32.1 to 45.8 (74)	-	14.7, 10.3 to 20.4 (28)	57.6, 50.5 to 64.4 (110)	-
Alcohol misuse (2706)	36.1, 34.3 to 37.9 (976)	1.0, 0.7 to 1.5 (27)	8.8, 7.8 to 9.9 (238)	64.5, 62.6 to 66.2 (1744)	19.1, 17.7 to 20.6 (518)
Alcohol disorder (1305)	37.7, 35.1 to 40.4 (492)	2.1, 1.4 to 3.0 (27)	7.5, 6.2 to 9.1 (98)	66.4, 63.8 to 68.9 (866)	19.7, 17.6 to 21.9 (257)
Substance misuse/disorder (1225)	41.1, 38.3 to 43.8 (503)	1.1, 0.7 to 1.9 (14)	7.8, 6.5 to 9.5 (96)	53.1, 50.3 to 55.9 (651)	17.8, 15.8 to 20.0 (218)
Multisubstance use (888)	41.3, 38.1 to 44.6 (367)	1.5, 0.9 to 2.5 (13)	7.4, 5.9 to 9.4 (66)	50.0, 46.7 to 53.3 (444)	15.0, 12.8 to 17.5 (133)
Personality disorder (1133)	48.2, 45.3 to 51.1 (546)	1.8, 1.1 to 2.7 (20)	8.6, 7.1 to 10.3 (97)	44.7, 41.8 to 47.6 (506)	5.1, 4.0 to 6.6 (58)
Learning difficulties or autism (241)	38.2, 32.3 to 44.5 (92)	4.2, 2.2 to 7.5 (10)	5.4, 3.1 to 9.1 (13)	51.0, 44.7 to 57.3 (123)	10.0, 6.8 to 14.2 (24)

− denotes low cell count.

* Individuals could be referred to more than one service, with the exception of the ‘GP only’ category where we excluded those with referrals to specialist mental health services, social services or VCSE organisation.

† Data on ethnic group were missing for n=565; data on IMD score were missing for ; data on IMD score were missing for ; of Manchester was ranked as the 4th most deprived Local Authority in England;-- denotes low cell count.

‡ Data on IMD score were missing for n=1499.

§ Data on IMD score were missing for n=1171.

¶ The City of Manchester was ranked as the fourth most deprived local authority in England.

GPgeneral practitionerIMDIndex of Multiple DeprivationVCSEvoluntary, community and social enterprise

### Mental health and social needs, care gaps and patient characteristics

The majority (96.2%, 25 893/26 909) of individuals were rated as having mental health needs (table 3). While proportions were high (90% or greater) in all groups, men, those aged 25 years or over and those who were unemployed were more likely to have mental health needs (table 3).

**Table 3:** Estimated mental health and social needs by groups of individuals (objective 3) (N=25 893 unless stated)

	Significant mental health needs (n/N)	%, 95% CI	Significant social needs (n/N)	%, 95% CI
Total	25 893/26 909	96.2 (96.0 to 96.4)	14 219/26 909	52.8 (52.2 to 53.4)
Women	14 347/15 019	95.5 (95.2 to 95.8)	7727/15 019	51.5 (50.6 to 52.2)
Men	11 546/11 890	97.1 (96.8 to 97.4)	6492/11 890	54.6 (53.7 to 55.5)
Age group				
15–19	3618/3931	92.0 (91.1 to 92.8)	2176/3931	55.4 (53.8 to 56.9)
20–24	4641/4874	95.2 (94.6 to 95.8)	2723/4874	55.9 (54.5 to 57.3)
25–34	6772/6982	97.0 (96.6 to 97.4)	3751/6982	53.7 (52.6 to 54.9)
35–44	5601/5749	97.4 (97.0 to 97.8)	3020/5749	52.5 (51.2 to 53.8)
45–64	4854/4954	98.0 (97.6 to 98.3)	2410/4954	48.7 (47.3 to 50.0)
65+	407/419	97.1 (95.0 to 98.4)	139/419	33.2 (28.8 to 37.8)
Ethnic group(N=26 344)				
White	22 643/23 421	96.7 (96.4 to 96.9)	12 322/23 421	52.6 (52.0 to 53.2)
Black	659/695	94.8 (92.9 to 96.2)	407/695	58.6 (54.9 to 62.2)
Indian/Pakistani/Bangladeshi	1079/1193	90.4 (88.6 to 92.0)	582/1193	48.8 (46.0 to 51.6)
Mixed race	506/521	97.1 (95.3 to 98.3)	295/521	56.6 (52.3 to 60.8)
Chinese	–	–	39/73	53.4 (42.0 to 64.5)
Other	–	–	267/441	60.5 (55.9 to 65.0)
Employment status (N=25 410)				
In work or study	9101/9616	94.6 (94.2 to 95.1)	4990/9616	51.9 (50.9 to 52.9)
Unemployed	11 308/11 585	97.6 (97.3 to 97.9)	6607/11 585	57.0.4 (56.1 to 57.9)
Registered sick	2463/2504	98.4 (97.8 to 98.8)	1195/2504	47.7.4 (45.8 to 50.0)
Retired	594/613	96.9 (95.2 to 98.0)	206/613	33.6 (30.0 to 37.4)
Looking after the home or family/other	1027/1092	94.1 (92.5 to 95.3)	484/1092	44.3 (41.4 to 47.3)
Area level deprivation (IMD quintile) (N=25 738)				
1 (least deprived)	4907/5065	96.9 (96.4 to 97.3)	2594/5065	51.2 (49.8 to 52.6)
2	4979/5178	96.2 (95.6 to 96.6)	2703/5178	52.2 (50.8 to 53.6)
3	4916/5151	95.4 (94.8 to 96.0)	2735/5151	53.1 (51.7 to 54.4)
4	4872/5034	96.8 (96.3 to 97.2)	2554/5034	50.7 (49.4 to 52.1)
5 (most deprived)	5092/5310	95.9 (95.3 to 96.4)	2796/5310	52.7 (51.3 to 54.0)
Primary psychiatric diagnosis				
None recorded	N/A	N/A	6588/12 746	51.7 (50.8 to 52.6)
Mood disorder	N/A	N/A	2377/4445	53.5 (52.0 to 54.9)
Psychotic disorder	N/A	N/A	245/613	40.0 (36.2 to 43.9)
Anxiety or trauma-related disorder	N/A	N/A	761/1416	53.7 (51.1 to 56.3)
Eating disorder	N/A	N/A	102/191	53.4 (46.3 to 60.4)
Alcohol misuse	N/A	N/A	1436/2706	53.1 (51.2 to 54.9)
Alcohol disorder	N/A	N/A	661/1305	50.7 (47.9 to 53.4)
Substance misuse/disorder	N/A	N/A	754/1225	61.6 (58.8 to 64.2)
Multisubstance use	N/A	N/A	554/888	62.4 (59.2 to 65.5)
Personality disorder	N/A	N/A	598/1133	52.8 (49.9 to 55.7)
Learning difficulties or autism	N/A	N/A	143/241	59.3 (53.0 to 65.4)

N/A: due to all people with psychiatric diagnosies having mental health needs.

– denotes cell counts too low to present data.

N/Anot available

Among the groups identified as having mental health needs, 29.9% (6503/21719) had no active or new referral to mental health services (table 4A). Proportions of non-referral were higher among men (33.7% vs 29.8% in women, aRR 1.14, 95% CI 1.09 to 1.18), younger people (eg, 42.5% among ages 15–19 years vs 24.1% for ages 65+, aRR 1.81, 95% CI 1.47 to 2.23), people from a black ethnic group (42.3% vs 30.8% among people from a white ethnic group, aRR 1.42, 95% CI 1.29 to 1.57), Indian/Pakistani/Bangladeshi groups (39.5%, aRR 1.32, 95% CI 1.21 to 1.43) and Chinese ethnic group (59.1%, aRR 2.09, 95% CI 1.68 to 2.59) (table 3 and table 4A). Within the group identified as having mental health needs, we also observed higher rates of non-referral among people living in areas in the most deprived quintile (34.7% vs 30.5% in the least deprived quintile, aRR 1.09, 95% CI 1.03 to 1.17). People with a mental health diagnosis of any type had higher rates of referral than those without a recorded diagnosis (of which 40.6% had no active or new referral). Within the group who had a mental health diagnosis, people with alcohol and substance misuse disorders had higher non-referral rates than those with other diagnoses (eg, alcohol misuse, 31.6% were not referred), as did people with an anxiety or trauma-related disorder (36.9%).

**Table 4:** Factors associated with non-referral among people with (A) mental health needs and (B) social needs: risk ratios and 95% CIs (objective 4)

(A) People with mental health needs			
	% with mental health needs who had no new or active referral (n/n)	Unadjusted RR (95% CI)	Adjusted RR (95% CI)
Total	29.9 (6503/21 719)		
Gender (N=21 719)			
Men	31.8 (3046/9578)	1.12 (1.07 to 1.16)	1.14 (1.09 to 1.18)
Women	28.5 (3457/12 141)	1	1
Age group (N=21 719)*			
15–19	40.6 (1272/3137)	1.86 (1.51 to 2.28)	1.81 (1.47 to 2.23)
20–24	33.9 (1342/3954)	1.55 (1.26 to 1.91)	1.53 (1.25 to 1.89)
25–34	29.3 (1632/5579)	1.39 (1.09 to 1.65)	1.30 (1.06 to 1.60)
35–44	27.0 (1236/4686)	1.24 (1.00 to 1.52)	1.17 (0.95 to 1.44)
45–64	22.5 (948/4140)	1.05 (0.85 to 1.29)	0.98 (0.80 to 1.21)
65	21.9 (73/334)	1	1
Ethnic group (N=21 230)*			
White	29.0 (5452/18 816)	1	1
Black	41.3 (239/579)	1.42 (1.29 to 1.57)	1.42 (1.29 to 1.57)
Indian/Pakistani/Bangladeshi	38.7 (368/951)	1.34 (1.23 to 1.45)	1.32 (1.21 to 1.43)
Mixed race	28.0 (128/458)	0.96 (0.83 to 1.12)	0.98 (0.85 to 1.14)
Chinese	61.8 (34/55)	2.13 (1.73 to 2.63)	2.09 (1.68 to 2.59)
Other	33.4 (124/371)	1.15 (1.00 to 1.33)	1.18 (1.02 to 1.36)
Employment status (N=20 419)*			
In work or study	37.2 (2935/7897)	1	1
Unemployed	26.2 (2467/9421)	0.70 (0.67 to 0.74)	0.71 (0.68 to 0.74)
Registered sick	19.9 (355/1786)	0.53 (0.49 to 0.59)	0.51 (0.46 to 0.56)
Retired	23.2 (113/488)	0.62 (0.53 to 0.73)	0.62 (0.53 –to 0.73)
Looking after the home or family/other	34.3 (284/827)	0.92 (0.84 to 1.02)	0.89 (0.81 to 0.99)
Area level deprivation (IMD quintile) (N=20 783)*			
1 (least deprived)	29.5 (1260/4270)	1	1
2	27.9 (1169/4189)	0.95 (0.88 to 1.01)	0.97 (0.91 to 1.04)
3	28.5 (1165/4088)	0.97 (0.90 to 1.03)	0.97 (0.91 to 1.04)
4	29.6 (1225/4137)	1.00 (0.94 to 1.07)	1.01 (0.95 to 1.08)
5 (most deprived)	32.5 (1334/4099)	1.10 (1.03 to 1.18)	1.09 (1.03 to 1.17)
Primary psychiatric diagnosis (N=21 719)			
None recorded	38.5 (3784/9819)	1	1
Mood disorder	15.6 (584/3737)	0.41 (0.37 to 0.44)	0.43 (0.40 to 0.47)
Psychotic disorder	3.8 (19/505)	0.10 (0.06 to 0.15)	0.11 (0.07 to 0.18)
Anxiety or trauma-related disorder	34.4 (348/1012)	0.89 (0.82 to 0.98)	0.87 (0.80 to 0.94)
Eating disorder	11.5 (19/165)	0.30 (0.20 to 0.46)	0.33 (0.21 to 0.50)
Alcohol misuse	30.8 (735/2390)	0.80 (0.75 to 0.85)	0.82 (0.77 to 0.87)
Alcohol disorder	27.2 (279/1026)	0.71 (0.64 to 0.78)	0.73 (0.66 to 0.80)
Substance misuse/disorder	32.1 (349/1086)	0.83 (0.76 to 0.91)	0.98 (0.89 to 1.07)
Multisubstance use	29.7 (240/809)	0.77 (0.69 to 0.85)	0.89 (0.79 to 0.99)
Personality disorder	11.4 (110/963)	0.30 (0.25 to 0.35)	0.34 (0.29 to 0.41)
Learning difficulties or autism	17.4 (36/207)	0.45 (0.34 to 0.61)	0.56 (0.41 to 0.75)

(B) People with social needs			
	% with social needs who had no referral to social or VCSE services (n/n)	Unadjusted RR (95% CI)	Adjusted RR (95% CI)
Total	79.6 (9469/11 892)		
Gender (N=11 892)*			
Men	82.3 (4439/5397)	1.06 (1.04 to 1.08)	1.06 (1.04 to 1.08)
Women	77.4 (5030/6495)	1	1
Age group (N=11 892)†			
15–19	70.7 (1301/1841)	0.92 (0.83 to 1.02)	0.94 (0.85 to 1.05)
20–24	77.1 (1763/2287)	1.00 (0.90 to 1.11)	1.03 (0.93 to 1.14)
25–34	81.9 (2536/3095)	1.06 (0.96 to 1.17)	1.09 (0.99 to 1.21)
35–44	82.6 (2065/2500)	1.07 (0.97 to 1.18)	1.10 (0.99 to 1.21)
45–64	83.5 (1716/2055)	1.08 (0.98 to 1.20)	1.11 (1.00 to 1.21)
65	77.2 (88/114)	1	1
Ethnic group (N=11 608)*			
White	79.7 (8140/10 213)	1	1
Black	76.5 (273/357)	0.96 (0.91 to 1.02)	0.96 (0.91 to 1.02)
Indian/Pakistani/Bangladeshi	79.3 (399/503)	1.00 (0.95 to 1.04)	1.00 (0.96 to 1.05)
Mixed race	83.6 (224/268)	1.05 (0.99 to 1.11)	-
Chinese	78.1 (25/32)	0.98 (0.82 to 1.18)	-
Other	83.0 (195/235)	1.04 (0.98 to 1.10)	-
Employment status (N=11 204)*			
In work or study	79.2 (3409/4305)	1	1
Unemployed	82.0 (4515/5508)	1.04 (1.01 to 1.06)	1.03 (1.01 to 1.05)
Registered sick	68.1 (572/840)	0.86 (0.82 to 0.90)	0.87 (0.83 to 0.91)
Retired	79.5 (132/166)	1.00 (0.93 to 1.09)	0.99 (0.92 to 1.08)
Looking after the home or family/other	70.1 (270/385)	0.89 (0.83 to 0.95)	0.88 (0.83 to 0.95)
Area level deprivation (IMD quintile) (N=11 205)*			
1 (least deprived)	81.8 (1839/2249)	1	1
2	79.0 (1788/2264)	0.97 (0.94 to 0.99)	0.99 (0.96 to 1.02)
3	79.8 (1816/2277)	0.98 (0.95 to 1.00)	1.01 (0.98 to 1.03)
4	79.5 (1721/2166)	0.97 (0.94 to 1.00)	0.99 (0.97 to 1.02)
5 (most deprived)	77.5 (1742/2249)	0.95 (0.92 to 0.98)	0.98 (0.95 to 1.01)
Primary psychiatric diagnosis (N=11 892)‡			
None recorded	76.9 (4135/5375)	1	1
Mood disorder	79.8 (1617/2026)	1.04 (1.01 to 1.07)	1.03 (1.00 (1.06)
Psychotic disorder	85.9 (165/192)	1.12 (1.05 to 1.19)	1.11 (1.04 to 1.17)
Anxiety or trauma-related disorder	66.6 (380/571)	0.87 (0.81 to 0.92)	0.86 (0.81 to 0.91)
Eating disorder	79.5 (66/83)	1.03 (0.93 to 1.15)	1.04 (1.06 to 1.12)
Alcohol misuse	83.9 (1077/1284)	1.09 (1.06 to 1.12)	1.09 (1.06 to 1.12)
Alcohol disorder	83.3 (454/545)	1.08 (1.04 to 1.13)	1.07 (1.03 to 1.12)
Substance misuse/disorder	87.6 (595/679)	1.14 (1.10 to 1.18)	1.13 (1.09 to 1.17)
Multisubstance use	87.9 (442/503)	1.14 (1.10 to 1.18)	1.14 (1.10 to 1.18)
Personality disorder	84.3 (428/508)	1.10 (1.05 to 1.14)	1.08 (1.04 to 1.13)
Learning difficulties or autism	87.3 (110/126)	1.13 (1.06 to 1.21)	1.12 (1.05 to 1.20)

Adjusted RRs adjusted for year of presentation, hour of presentation, hospital attended, role of assessor (doctor or nurse) and method of harm.Not adjusted for hour or year of presentation due to model nonconvergence

– denotes cell counts too low to estimate adjusted RR.

* Not adjusted for hour or year of presentation due to model non-convergence.

† Not adjusted for year of presentation or hospital attended due to model non-convergence.

‡ Not adjusted for hour of presentation, hospital attended or method of harm due to model non-convergence.

IMDIndex of Multiple DeprivationRRrisk ratioVCSEvoluntary, community and social enterprise

Just over half 52.8% (14 219/26 909) of individuals were estimated as having social needs. Men, those aged under 35, people from a black ethnic group, those who were unemployed and people with a substance misuse disorder were more likely to have social needs (table 3).

Among people with social needs, 79.6% (9469/11 892) had no new referral to social and/or VCSE services (table 4B). 23.0% (3269/14 219) also had no active or new referral to mental health services. Proportions of those with no new referral to social and/or VCSE services among those with identified social needs were higher for men (82.3% vs 77.4% among women, aRR 1.06, 95% CI 1.04 to 1.08), people aged 45–64 (83.5% vs 77.2% among 65+ years, aRR 1.11, 95% CI 1.00 to 1.21) and those who were unemployed 82.0% vs 79.9% among those in work or study, aRR 1.03, 95% CI 1.01 to 1.05). With the exception of anxiety and trauma-related disorders, individuals with a mental health diagnosis who had social needs had higher rates of non-referral than those with no recorded diagnosis (table 4B). People with substance misuse disorders who had social needs had especially high rates of non-referral: substance misuse disorder 87.6%, aRR 1.13, 95% CI 1.09 to 1.17 and multisubstance misuse aRR 87.9%, 1.14, 95% CI 1.10 to 1.18.

## Discussion

### Main findings

The majority of individuals were estimated as having mental healthcare needs and just over half of individuals were estimated as having significant social needs. In terms of care gaps, almost one-third of people presenting to the ED following self-harm who had mental health needs had no new or active referral to mental health services. For people with social needs, the care gap was substantially larger, with 8 in 10 having no new referral to social or VCSE services. The mental healthcare gap was higher for men, younger people, those from a black, South Asian or Chinese ethnic group, those from the most deprived areas, those with no mental health diagnosis and those with an alcohol or substance misuse disorder, or an anxiety or trauma-related disorder. Among individuals with social needs, the care gap (ie, no new referral to social and/or VCSE services) was higher for men, individuals aged 45–64, those who were unemployed and those with a diagnosed mental disorder (particularly substance misuse).

### Strengths and limitations

This is the first study of referrals to mental health, social and VCSE services and GP care and care gaps for people attending hospital following self-harm. The use of a self-harm cohort study allowed a detailed assessment of patients’ needs, beyond the basic patient measures which are commonly recorded in electronic health records. The main limitation is that we could not include people who did not receive a psychosocial assessment because the information relating to mental health and social needs was not available in this group. Non-assessment has been found to be associated with some indicators of need, including having engaged in substance or alcohol misuse at the time of self-harm.^24^ As a consequence, our study is likely to underestimate the needs of people presenting to the hospital after self-harm (though mental health needs were consistently high at around 95%). We were able to include self-harm presentations up to 2017 only, due to the availability of data. The single-centre cohort, based in a relatively socioeconomically deprived area of England, may not be representative of the broader population of people presenting to the hospital following self-harm.

The use of established measurement scales would have provided more accurate and nuanced measures of mental health and social needs; for example, we were unable to estimate the severity of needs or discern the level of impairment to daily activities. In addition, there is likely to be some overlap between mental health and social needs, with some mental health needs potentially met by social care and VCSE services and vice versa. Finally, people may have been receiving help from sources not recorded in the study, for example, from private or workplace therapy, from family and friends or from other services.

While we were able to obtain information about existing mental health services and GP care, we were not able to ascertain if people were already receiving input from social services. We did not include people receiving current treatment for mental health as experiencing unmet mental health needs as we concurred that this indicated their needs would be met, though we acknowledge that a current or new referral to services does not necessarily mean that an individual receives appropriate care or any care. Barriers such as long waiting times and referrals being rejected by the service can contribute to people experiencing exclusion from follow-up services.^9 25^ Finally, we acknowledge that patients seeking help from an ED following self-harm represent the tip of the iceberg of all self-harm, due to a substantial proportion of people not seeking help.^26^

### Comparison with existing evidence

Care gaps for mental health in our study were greater in ethnic minority groups. We also found that black and South Asian groups were more likely to be referred solely to their GP for mental healthcare. Previous research has found that people from ethnic minority groups who died by suicide were more likely to be unemployed, to live in unstable housing and to live in areas of higher deprivation.^27^ Individuals from ethnic minority groups were also viewed as lower risk and were less likely to receive certain types of care such as crisis home treatment services. We have shown that, among ethnic minority groups presenting to the hospital for self-harm, not only are levels of social adversity higher, but the care gap is greater. Approaches to reducing ethnic group inequalities in access to mental healthcare include reverse commissioning, training for care providers to deliver more culturally sensitive services and interactions and patient and public involvement of people from ethnic minority groups in designing service provision.^28^

We also found elevated care gaps for individuals with social needs among middle-aged men, a group previously identified as at particular risk of experiencing socioeconomic adversity.^29^ Socioeconomic difficulties are also strongly associated with suicide in midlife.^30^ Our findings suggest that social problems in midlife are accompanied by comparatively low levels of follow-up support for people who have self-harmed. This is particularly important considering the relatively high suicide rates in this age group.^1^

In an example of the inverse care law,^31^ previous research has identified that the probability of mental health services referrals following self-harm is lower for people in more deprived neighbourhoods and that rates of self-harm are higher in those same neighbourhoods.^32 33^ While studies have attempted to explain the associations between area-level characteristics and self-harm rates,^34 35^ our research provides insight at the individual level. While we did not find lower referral rates among people from areas of higher deprivation, we found that the gap between mental health needs and the likelihood of referral was greater for people living in the most deprived areas. In other words, the mental healthcare gap was greater for people in more deprived neighbourhoods seeking help for self-harm.

We found evidence of mental healthcare needs in the majority of individuals. In a systematic review, 84% of adults presenting to the hospital for self-harm had at least one psychiatric disorder when assessed using a range of diagnostic tools.^36^ This suggests our estimate of mental health needs in this population is plausible. However, we acknowledge there is uncertainty around our estimate. Previous research has indicated that people who had no diagnosed mental illness had especially low rates of psychosocial assessment and mental health services referral following self-harm.^37^ In our study, the mental healthcare gap was greater among people with no diagnosed mental health condition. Individuals with no diagnosis were more likely to be referred solely to their GP for mental health support. Our findings imply that the absence of a diagnosed mental disorder among people seeking help following self-harm could act as a barrier to accessing aftercare for those with mental healthcare needs. This finding is consistent with qualitative research on patient and staff experiences of accessing self-harm aftercare.^9 25^ We also found lower levels of referrals to social and VCSE services alongside greater social needs among people with a mental health diagnosis, with greater care gaps for those with a substance misuse diagnosis. Substance misuse has previously been linked to lower likelihood of referral in episodes of self-poisoning^38^ and exclusion from mental health services.^39^ Research has suggested referrals alone are not sufficient for this group—active follow-up helping to link individuals to services following the referral is recommended.^40^

### Implications for practice and research

Two key recommendations for hospital presentations involving self-harm are psychosocial assessment by a mental health specialist and to consider referral for psychological therapy.^2^ Our findings suggest that the provision of recommended care is not proportionate to need, with men, younger people, those from a black, South Asian or Chinese ethnic group, those from the most deprived areas and those with an alcohol, substance misuse or anxiety or trauma-related disorder having lower levels of access to potentially effective treatments. Efforts to increase the provision of mental health support should be targeted towards these groups in particular.

The considerable gaps in access to social and VCSE services identified in this study underline the importance of involving non-health sector professionals in developing treatment plans and conducting psychosocial assessments. A recent review found evidence that non-clinical self-harm services were viewed more positively than clinical services.^41^ However, people reported being unsure of which non-clinical services were available to them, in part due to poor integration between social/voluntary services and clinical services.

Future research should focus on integrated approaches to self-harm care. Systems approaches to suicide prevention show promise, particularly multicomponent models and those that are tailored to specific needs of communities.^42^ Developing new models of integrated care between primary, secondary and VCSE services is a key objective of the Community Mental Health Framework in England.^43^ This initiative has the potential to reduce inequities in access to mental health and social support. For example, the 42 Integrated Care Systems across England are currently being supported to develop codesigned, evidence-based interventions and reduce fragmentation between services for people who have self-harmed.^1 44^ Investment in aftercare for individuals seeking help for self-harm is vital for addressing the high risks of suicide in this group.^45^

## Conclusions

We found substantial care gaps among people presenting to the hospital following self-harm, with particularly large gaps for individuals with social needs. Care gaps were particularly high among groups known to be at increased risk of suicide: men, those in middle age, unemployed individuals and those with a substance misuse disorder. The greater mental healthcare gaps in ethnic minority groups suggest services are not adequately recognising and actioning appropriate aftercare following self-harm. Training and support for health and social care providers to engage with people from ethnic minority groups to help develop appropriate services is recommended. The role of social and VCSE services in self-harm aftercare is only recently being prioritised in suicide prevention policy. Our findings suggest this is a key area for closing the gaps and reducing inequalities in self-harm aftercare. Improving links between health, social and VCSE services is vital in achieving this.

## supplementary material

10.1136/bmjopen-2024-085672 online supplemental file 1

10.1136/bmjopen-2024-085672 online supplemental file 2

## Data Availability

No data are available.
