# Supplementary material for: Care gaps among people presenting to the hospital following self-harm: observational study of three emergency departments in England
Source: BMJ Open. 2024 Oct 22;14(10):e085672. doi: 10.1136/bmjopen-2024-085672 (PMC11499793; doi:10.1136/bmjopen-2024-085672)
Supplement: online supplemental file 1 [file bmjopen-14-10-s001.pdf]

Table S1: Proportions of missing data among assessed self-harm episodes by study population characteristics and study outcomes

	n (%)	n (%) with mental health needs	n (%) referred to mental health services
Total (26909)			
Ethnic group missing	565 (2.1)	532 (94.2)	198 (35.0)
Ethnic group not missing	26344 (97.9)	25361 (96.3)	9718 (36.9)
Employment status missing	1499 (5.6)	1400 (93.4)	588 (39.2)
Employment status not missing	25410 (94.4)	24493 (96.4)	9328 (36.7)
Area level deprivation missing	1171 (4.4)	1127 (96.2)	385 (32.9)
Area level deprivation not missing	25738 (95.6)	24766 (96.2)	9531 (37.0)
