# Supplementary material for: Care gaps among people presenting to the hospital following self-harm: observational study of three emergency departments in England
Source: BMJ Open. 2024 Oct 22;14(10):e085672. doi: 10.1136/bmjopen-2024-085672 (PMC11499793; doi:10.1136/bmjopen-2024-085672)
Supplement: online supplemental file 2 [file bmjopen-14-10-s002.pdf]

Table S2: Proportions of patients referred to mental health services, by type of service [a]

	**%, 95% CI (n) referred to mental health services**	- %, 95% CI (n) referred to outpatient mental health follow-up	- %, 95% CI (n) referred to crisis or urgent care services	- %, 95% CI (n) referred to community mental health services	- %, 95% CI (n) referred to drug and alcohol services
Total (26909)	36.9, 36.3 – 37.4 (9916)	13.2, 12.8 – 13.6 (3542)	9.8, 9.4 – 10.1 (2623)	3.5, 3.3 – 3.8 (948)	4.0, 3.8 – 4.2 (1072)
Women (15019)	35.5, 34.7 – 36.3 (5331)	13.9, 13.4 – 14.5 (2089)	9.7, 9.3 – 10.3 (1460)	3.8, 3.5 – 4.1 (570)	3.0, 2.7 – 3.2 (445)
Men (11890)	38.6, 37.7 – 39.4 (4585)	12.2, 1 1.6 – 12.8 (1453)	9.8, 9.3 – 10.2 (1163)	3.2, 2.9 – 3.5 (378)	5.3, 4.9 – 5.7 (627)
Age group					
15-19 (3931)	30.9, 29.4 – 32.3 (1213)	15.0, 13.9 – 16.1 (588)	8.7, 7.8 – 9.6 (340)	2.2, 1.8 – 2.8 (88)	--
20-24 (4874)	33.4, 32.1 – 34.7 (1626)	12.4, 11.5 – 13.3 (604)	10.2, 9.4 – 11.1 (497)	3.5, 3.0 – 4.1 (172)	2.3, 1.9 – 2.7 (110)
25-34 (6982)	38.1, 37.0 – 39.2 (2660)	13.0, 12.2 – 13.8 (904)	10.2, 9.5 – 10.9 (712)	3.7, 3.3 – 4.1 (256)	4.6, 4.2 – 5.2 (324)
35-44 (5749)	38.2, 37.0 – 39.5 (2196)	12.9, 12.1 – 13.8 (743)	8.3, 7.6 – 9.0 (476)	4.1, 3.6 – 4.6 (234)	6.1, 5.5 – 6.7 (349)
45-64 (4954)	39.8, 38.4 – 41.1) (1969)	13.0, 12.1 – 13.9 (642)	11.4, 10.5 – 12.2 (563)	3.7, 3.2 – 4.3 (183)	4.9, 4.3 – 5.5 (241)
65+ (419)	60.1, 55.4 – 64.7 (252)	14.6, 11.5 – 18.3 (61)	8.4, 6.1 – 11.4 (35)	3.5, 2.2 – 5.9 (948)	--
Ethnic group (26344) [1]					
White (23421)	36.9, 36.3 – 37.5 (8648)	13.3, 12.8 – 13.7 (3103)	9.5, 9.1 – 9.8 (2213)	3.6, 3.4 – 3.9 (844)	4.3, 4.0 – 4.6 (1004)
Black (695)	39.1, 35.6 – 42.8 (272)	16.0, 13.4 – 18.9 (111)	10.9, 8.8 – 13.5 (76)	2.9, 1.9 – 4.4 (20)	2.0, 1.2 – 2.1 (14)
Indian/Pakistani/Bangladeshi (1193)	34.0, 31.4 – 36.8 (406)	11.9, 10.2 – 13.9 (142)	10.3, 8.7 – 12.2 (123)	3.8, 2.8 – 5.0 (45)	1.3, 0.8 – 2.1 (15)
Mixed race (521)	41.1, 36.9 – 45.4 (214)	12.7 10.1 – 15.8 (66)	14.2, 11.5 – 17.5 (74)	--	--
Chinese (73)	26.0, 17.3 – 37.2 (19)	--	--	--	--
Other (441)	36.1, 31.7 – 40.6 (159)	14.1, 11.1 – 17.6 (62)	13.6, 10.7 – 17.1 (60)	--	--
Employment status [2] *(25410)*					
In work or study (9616)	31.3, 30.4 – 32.2 (3009)	12.5, 11.9 – 13.2 (1203)	9.8, 9.2 – 10.4 (940)	2.3, 2.1 – 2.6 (222)	2.3, 2.0 – 2.6 (223)
Unemployed (11585)	39.9, 39.0 – 40.8 (4623)	12.1, 11.5 – 12.7 (1398)	10.9, 10.3 – 11.5 (1262)	3.6, 3.3 – 4.0 (418)	5.4, 5.0 – 5.8 (624)
Registered sick (2504)	40.7, 38.8 – 42.6 (1019)	22.4, 20.8 – 24.0 (560)	3.7, 3.0 – 4.5 (93)	7.7, 6.7 – 8.8 (193)	5.4, 4.6 – 6.3 (135)
Retired (613)	53.8, 49.9 – 57.7 (330)	13.4, 10.9 – 16.3 (82)	10.6, 8.4 – 13.3 (65)	3.0, 6.3 – 13.3 (27)	2.3, 1.4 – 3.8 (14)
Looking after the home or family/other (1092)	31.8, 29.1 – 34.6 (347)	13.0, 11.1 – 15.1 (142)	7.3, 5.9 – 9.0 (80)	4.1, 3.1 – 5.5 (45)	3.1, 2.2 – 4.3 (34)
Area-level deprivation (IMD) quintile (25738) [± 3]					
1 (least deprived) (5065)	35.0, 33.7 – 36.3 (1773)	13.0, 12.1 – 13.9 (658)	9.8, 9.0 – 10.6 (495)	2.9, 2.5 – 3.4 (149)	2.6, 2.2 – 3.0 (130)
2 (5178)	38.8, 37.5 – 40.2 (2010)	13.7, 12.8 – 14.7 (709)	11.5, 10.7 – 12.4 (597)	4.0, 3.5 – 4.5 (205)	3.3, 2.9 – 3.9 (173)
3 (5151)	38.2, 36.9 – 39.5 (1968)	14.0, 13.1 – 15.0 (722)	9.8, 9.0 – 10.6 (505)	3.8, 3.4 – 4.4 (198)	4.5, 4.0 – 5.1 (231)
4 (5034)	39.0, 37.7 – 40.4 (1965)	14.1, 13.1 – 15.1 (708)	9.7, 8.9 – 10.5 (486)	3.6, 3.1 – 4.1 (181)	4.9, 4.4 – 5.6 (248)
5 (most deprived) (5310)	34.2, 32.9 – 35.5 (1815)	11.6, 10.8 – 12.5 (617)	9.1, 8.3 – 9.9 (482)	3.6, 3.1 – 4.2 (192)	4.4, 3.9 – 5.0 (235)
Primary psychiatric diagnosis (26909)					

None recorded (12746)	29.8, 29.0 – 30.6 (3799)	12.5, 11.9 – 13.1 (1587)	8.4, 7.9 – 8.9 (1067)	3.4, 3.1 – 3.7 (427)	1.1, 0.9 – 1.3 (142)
Mood disorder (4445)	49.2, 47.8 – 50.7 (2188)	17.4, 16.3 – 18.5 (773)	14.4, 13.4 – 15.5 (640)	4.2, 3.6 – 4.8 (185)	1.9, 1.5 – 2.3 (83)
Psychotic disorder (613)	68.0, 64.2 – 71.6 (417)	16.5, 13.7 – 19.6 (101)	14.4, 11.8 – 17.4 (88)	9.0, 7.0 – 11.5 (55)	--
Anxiety or trauma-related disorder (1416)	32.6, 30.2 – 35.1 (462)	13.9, 12.2 – 15.8 (197)	7.3, 6.1 – 8.8 (104)	6.1, 5.0 – 7.5 (87)	2.3, 1.6 – 3.2 (32)
Eating disorder (191)	38.7, 32.1 – 45.8 (74)	16.2, 11.7 – 22.2 (31)	13.1, 9.0 – 18.7 (25)	--	--
Alcohol misuse (2706)	36.1, 34.3 – 37.9 (976)	11.0, 9.9 – 12.2 (298)	7.7, 6.7 – 8.8 (208)	2.5, 2.0 – 3.2 (68)	13.5, 12.3 – 14.8 (365)
Alcohol disorder (1305)	37.7, 35.1 – 40.4 (492)	8.0, 6.6 – 9.6 (104)	3.9, 3.0 – 5.1 (51)	2.5, 1.7 – 3.4 (32)	19.2, 17.2 – 21.5 (251)
Substance misuse/disorder (1225)	41.1, 38.3 – 43.8 (503)	10.4, 8.8 – 12.2 (127)	14.4, 12.5 – 16.4 (176)	--	6.8, 5.5 – 8.3 (83)
Multi-substance use (888)	41.3, 38.1 – 44.6 (367)	11.5, 9.5 – 13.8 (102)	10.9, 9.0 – 13.2 (97)	1.9, 1.2 – 3.1 (17)	10.1, 8.3 – 12.3 (90)
Personality disorder (1133)	48.2, 45.3 – 51.1 (546)	16.3, 14.3 – 18.6 (185)	12.4, 10.6 – 14.4 (140)	4.2, 3.2 – 5.6 (48)	1.2, 0.7 – 2.1 (14)
Learning difficulties or autism (241)	38.2, 32.3 – 44.5 (92)	15.4, 11.3 – 20.5 (37)	11.2, 7.8 – 15.8 (27)	--	--

[a] Individuals could be referred to more than one service.

[1] Data on ethnic group were missing for n=565; [2] data on IMD score were missing for n=1499; [3] data on IMD score were missing for n=1171; [±] The City of Manchester was ranked as the 4th most deprived Local Authority in England; -- denotes low cell count
