# Supplementary material for: Care gaps among people presenting to the hospital following self-harm: observational study of three emergency departments in England
Source: BMJ Open. 2024 Oct 22;14(10):e085672. doi: 10.1136/bmjopen-2024-085672 (PMC11499793; doi:10.1136/bmjopen-2024-085672)
Supplement: Supplementary file 3 [file bmjopen-2024-085672.reviewer_comments.pdf]

PEER REVIEW HISTORY

BMJ Open publishes all reviews undertaken for accepted manuscripts. Reviewers are asked to complete a checklist review form and are provided with free text boxes to elaborate on their assessment. These free text comments are reproduced below.

ARTICLE DETAILS

**TITLE (PROVISIONAL)**	Care gaps among people presenting to hospital following self-harm: observational study of three emergency departments in England
**AUTHORS**	Steeg, Sarah; Bickley, Harriet; Clements, Caroline; Quinlivan, Leah; Barlow, Steven; Monaghan, Elizabeth; Naylor, Fiona; Smith, Jonathan; Mughal, Faraz; Robinson, Catherine; Gnani, Shamini; Kapur, Navneet

VERSION 1 - REVIEW

**REVIEWER NAME**	Luo, Yanan
**REVIEWER AFFILIATION**	Peking University, Department of Global Health
**REVIEWER CONFLICT OF INTEREST**	I have no competing interests.
**DATE REVIEW RETURNED**	03-Jul-2024

**GENERAL COMMENTS**	Comments to the Author<br>Manuscript ID: bmjopen-2024-085672<br>General comments: Many thanks for this interesting and much improved manuscript. The manuscript utilized hospital records data from EDs in Manchester to estimate the unmet mental need and social needs among people presenting to hospital after self-harm, with a large sample of 26,090 patients from 1997-2017. I have a few additional comments:<br>Methods:<br>- 1. The authors are recommended to incorporate models that adequately account for within-individual variation to avoid discarding additional data of the cohort study spanning from 1997-2017. Could the authors make it more explicit how the issue has been addressed?<br>- 2. The authors did not include people who have received current treatment for mental health, assuming that their needs have been met. From my perspective, this group of patients should be included as population whose mental health needs have been met even without referral care.<br>- 3. Care gaps were defined as “the percentage of individuals who require care but do not receive treatment” in the manuscript. However, there is no such treatments in aftercare. According to the reference cited “[24] Kohn, Robert, Saxena, Shekhar, Levav, Itzhak & Saraceno, Benedetto. (2004). The treatment gap in mental health care. Bulletin of the World Health Organization, 82 (11), 858 - 866. World Health Organization. https://iris.who.int/handle/10665/269274”, this definition “the percentage of individuals who require care but do not receive treatment” was used to define treatment gap. Estimating the treatment gap in a population depends on the prevalence period of the disorder (i.e., prevalence rate), the time frame of the examination of service utilization, and the demographic representativeness of the study sample with reference to the target

	population. A brief explanation of the term definition is needed or if the authors means the percentage of individuals who require care but do not receive (referral) care?<br>- 4. There are some concept, including clinical management/hospital management, treatment, referrals, care, aftercare, etc., which are suggested to be uniformed throughout the manuscript. Or more clarification of the difference in concept or terminology is needed.<br>- 5. Could the authors be more explicit about the measures used in the Methods as in the abstract ? In the manuscript, it was written as "Clinical management", "Accessing clinical management and mental health and social needs of patients" and "Additional study measures". In the abstract, it was clearly written in terms of the primary and secondary measures.<br>Results:<br>- 6. These could be more clearly stated. "Men, those who were younger, from a Black, South Asian or Chinese ethnic group, …… had greater mental health care gaps."might be corrected as "Men, those who were younger (except for 45-64), from a Black, South Asian or Chinese or other ethnic group, …… had greater mental health care gaps.".<br>- 7. If the estimates of MH needs are so consistently high (above 95%), is the MH care gap plausible? The authors explained this in the former response letter. Could the authors be more explicit about this issue and added it to the Discussion?

VERSION 1 – AUTHOR RESPONSE

Reviewer: 2

Dr. Yanan Luo, Peking University, Peking University

Comments to the Author:

Comments to the Author

Manuscript ID: bmjopen-2024-085672

General comments: Many thanks for this interesting and much improved manuscript. The manuscript utilized hospital records data from EDs in Manchester to estimate the unmet mental need and social needs among people presenting to hospital after self-harm, with a large sample of 26,090 patients from 1997-2017. I have a few additional comments:

Methods:

- 1. The authors are recommended to incorporate models that adequately account for within-individual variation to avoid discarding additional data of the cohort study spanning from 1997-2017. Could the authors make it more explicit how the issue has been addressed?

We have explained in the Methods section that:

'We analysed individuals rather than episodes due to many of the exposure characteristics (for example, gender, age, ethnic group, mental health diagnosis) being measured at an individual level. In addition, mental health care gaps are typically measured at the individual level [14]; including multiple episodes by the same individual would likely lead to an inaccurate estimation of care gaps.

Where there were multiple episodes by the same individual, the individual's first assessed episode during the study period was included.'

In addition, we added to Methods, Missing data section, page 10:

'Missing data on age, sex and ethnic group were imputed using data from any additional episodes from the same individual recorded in the Manchester Self-Harm Project dataset.'

- 2. The authors did not include people who have received current treatment for mental health, assuming that their needs have been met. From my perspective, this group of patients should be included as population whose mental health needs have been met even without referral care.

This is correct and reflects our approach as described in Discussion, page 21:

'We did not include people receiving current treatment for mental health as experiencing mental health needs as we concurred that this indicated their needs would be met, though we acknowledge that a current or new referral to services does not necessarily mean that an individual receives appropriate care or any care.'

We have clarified this in Methods, page 9, in case it was not clear:

'Individuals were defined as having mental health care needs met if they were currently receiving mental health care or were referred to mental health services following their hospital presentation for self-harm. Significant social needs were defined as being met if the individual was referred to social services or VCSE services.'

- 3. Care gaps were defined as "the percentage of individuals who require care but do not receive treatment" in the manuscript. However, there is no such treatments in aftercare. According to the reference cited "[24] Kohn, Robert, Saxena, Shekhar, Levav, Itzhak & Saraceno, Benedetto. (2004). The treatment gap in mental health care. Bulletin of the World Health Organization, 82 (11), 858 - 866. World Health Organization. https://iris.who.int/handle/10665/269274 [iris.who.int]", this definition "the percentage of individuals who require care but do not receive treatment" was used to define treatment gap. Estimating the treatment gap in a population depends on the prevalence period of the disorder (i.e., prevalence rate), the time frame of the examination of service utilization, and the demographic representativeness of the study sample with reference to the target population. A brief explanation of the term definition is needed or if the authors means the percentage of individuals who require care but do not receive (referral) care?

We added to the above definition 'the percentage of individuals who require care but do not receive treatment' the following text to Methods, page 10:

'…with the term 'treatment' encompassing existing treatment and new referrals to care made following the hospital presentation.'

We made the assumption that referral to aftercare led to treatment, but also acknowledged that referrals may not always lead to treatment. Discussion, page 21:

'we acknowledge that a current or new referral to services does not necessarily mean that an individual receives appropriate care or any care. Barriers such as long waiting times and referrals being rejected by the service can contribute to people experiencing exclusion from follow-up services [9, 26].'

We have added to the Strengths and Limitations section, Discussion, page 21:

'The single-centre cohort, based in a relatively socioeconomically deprived area of England, may not be representative of the broader population of people presenting to hospital following self-harm.'

- 4. There are some concept, including clinical management/hospital management, treatment, referrals, care, aftercare, etc., which are suggested to be uniformed throughout the manuscript. Or more clarification of the difference in concept or terminology is needed.

When referring to hospital/clinical management we now refer to 'hospital management'.

We have defined the term 'treatment', in the context of treatment gaps, on page 10 as:

'… encompassing existing treatment and new referrals to care made following the hospital presentation.'

However, as noted in Methods (Page 5): 'In the present study we use the term 'care gaps'; this concept has been recommended as more appropriate for mental health as it takes into account non-clinical interventions and psychosocial needs'.

We now use the term 'care' to refer to health care services (e.g. mental health treatment) and social and VCSE care.

- 5. Could the authors be more explicit about the measures used in the Methods as in the abstract ? In the manuscript, it was written as “Clinical management”, “Accessing clinical management and mental health and social needs of patients” and “Additional study measures”. In the abstract, it was clearly written in terms of the primary and secondary measures.

We now use the following subheadings in the Methods:

Clinical management (secondary outcome measures)

Care gaps (primary outcome measures)

Study covariates

Results:

- 6. These could be more clearly stated. “Men, those who were younger, from a Black, South Asian or Chinese ethnic group, …… had greater mental health care gaps.”might be corrected as “Men, those who were younger (except for 45-64), from a Black, South Asian or Chinese or other ethnic group, …… had greater mental health care gaps.”.

We have amended this (Page 1) to:

‘Mental health care gaps were greater in men and those who were aged under 35 years, from a Black, South Asian or Chinese ethnic group, living in the most deprived areas, and had no mental health diagnosis, or an alcohol, substance misuse, anxiety or trauma-related disorder.’

- 7. If the estimates of MH needs are so consistently high (above 95%), is the MH care gap plausible? The authors explained this in the former response letter. Could the authors be more explicit about this issue and added it to the Discussion?

We have added the following to the Discussion, Page 23:

‘We found evidence of mental health care needs in the majority of individuals. In a systematic review, 84% of adults presenting to hospital for self-harm had at least one psychiatric disorder, when assessed using a range of diagnostic tools [36]. This suggests our estimate of mental health need in this population is plausible. However, we acknowledge there is uncertainty around our estimate.’
